# Impact of Mannose-Binding Lectin 2 Polymorphism on the Risk of Hepatocellular Carcinoma: A Case-Control Study in Chinese Han Population

**DOI:** 10.2188/jea.JE20140194

**Published:** 2015-05-05

**Authors:** Yong Lin, Chenghao Su, Jianjun Niu, Zhinan Guo, Lin Cai

**Affiliations:** 1 School of Public Health, Fujian Medical University, Fuzhou, Fujian Province, People’s Republic of China; 2 Office for Director, Xiamen Center for Disease Control and Prevention, Xiamen, Fujian Province, People’s Republic of China; 3 Zhongshan Hospital, Xiamen University, Fujian Province, Xiamen, People’s Republic of China; 4 Deparment of Vector-borne Disease Control, Xiamen Center for Disease Control and Prevention, Xiamen, Fujian Province, People’s Republic of China

**Keywords:** mannose-binding lectin 2, polymorphism, case-control study, hepatocellular carcinoma

## Abstract

**Background:**

Mannose-binding lectin2 (MBL2) is implicated in the host immune response, but there are limited data about *MBL2* polymorphisms and hepatocellular carcinoma (HCC) risk. This study aimed to investigate the relationship between the *MBL2* rs7096206 polymorphism and HCC risk in a Chinese Han population.

**Methods:**

A population-based case-control study of 220 HCC patients and 220 age- and gender-matched healthy control subjects from a Chinese Han population was conducted. Genomic DNA was extracted from blood samples, and the presence of the *MBL2* polymorphism rs7096206 was assessed using matrix-assisted laser desorption-ionization time-of-flight mass spectrometry. Conditional logistic regression was performed to assess the risk of HCC by determining odds ratios and 95% confidence intervals (CIs).

**Results:**

The odds of HCC among carriers of CG and GG genotypes were 7.33 (95% CI, 2.53–21.29) and 12.48 (95% CI, 2.08–74.90), respectively. In the dominant genetic model, GG+CG carriers had an approximately 8-fold increased risk (95% CI, 2.83–22.62) compared with those with the CC genotype. The G allele was significantly associated with elevated HCC risk, with an odds ratio of 6.83 (95% CI, 2.90–16.10).

**Conclusions:**

Our findings suggest that the *MBL2* polymorphism rs7096206 is associated with HCC susceptibility and has the potential to serve as a biomarker to detect populations at increased HCC risk.

## INTRODUCTION

Hepatocellular carcinoma (HCC) is currently an international public health issue because of its high prevalence and poor survival rate. It also places a heavy economic burden on individuals and health care systems. According to global cancer statistics released in 2011, HCC ranked fifth in global incidence and second in the number of cancer deaths.^1^ Chronic hepatitis B virus (HBV) infection is the major risk factor for HCC development, being responsible for approximately 60% of HCC cases in HBV-epidemic areas and 23% in developed countries.^2^

The HBV vaccine is an effective way to prevent HBV infection and subsequent HCC. With the application of the universal infant hepatitis vaccination program, the proportion of HBV-induced HCC cases is likely to continue to fall, and total incidence should also decline over time. The supply of clean water, health promotion, and anti-viral therapy can also help to limit the damage caused by HCC. However, the incidence of HCC is increasing in some countries, possibly as a result of an obesity epidemic or of hepatitis C virus (HCV) infection resulting from injection drug use.^3^

The human mannose-binding lectin (MBL) 2 gene (*MBL2*), which is composed of four exons (Figure), is located on chromosome 10q11.2-q21^4^ and plays a critical role in the innate immune system. Individual variations in serum MBL levels are mainly attributed to the presence of structural mutations in the MBL2 protein. The rs7096206 single nucleotide polymorphism (SNP) is located in the *MBL2* promoter region and involves a single C to G nucleotide substitution. Liver cells transfected with the rs7096206G variant were previously shown in functional promoter analyses to exhibit low promoter activity and, consequently, lower serum MBL2 levels.^5^ Low or defective MBL production could compromise the activation of the complement system, and carcinogenesis is possible under such circumstances. Recently, several multi-center studies with large sample sizes have revealed associations between other SNPs and HCC risk, while *MBL2* polymorphisms have been associated with leprosy in the Han Chinese population.^6^ Moreover, a study has been conducted assessing the association between *MBL2* polymorphisms and HCC,^7^ although the sample size was relatively small and no data about HBV-free populations were available. Therefore, the present case-control study investigated the role of the *MBL2* rs7096206 polymorphism on HCC risk in a Han Chinese population with no HBV infection.

**Figure.**
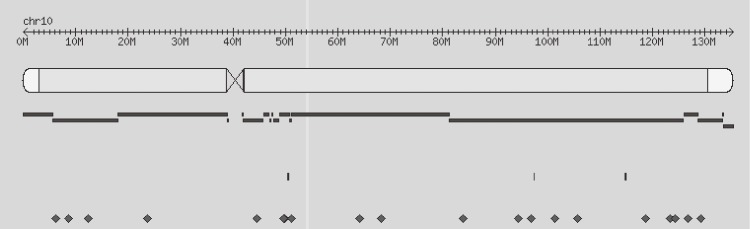
Physical location of rs7096206 in chromosome 10. rs7096206 is located at the -221 position of the MBL2 promoter region (Chr10:54531685); the closest gene next to MBL2 (from 5-3 direction) is small nuclear ribonucleoprotein polypeptide E pseudogene 8 (SNRPEP8), which has no function.

## MATERIALS AND METHODS

### Study subjects

Han Chinese patients with newly diagnosed HCC (*n* = 220) were recruited from the Xiamen Traditional Medicine Hospital and Xiamen Zhongshan Hospital between November 2011 and August 2013. Histological or cytological specimens were available from all recruited HCC cases. Patients were excluded if any of the following conditions were met: (1) HBV or HCV infection; (2) liver disease due to parasitosis, diabetes, fatty liver, metabolism disorders, or severe cardiovascular diseases; (3) presence of tumors other than HCC; (4) autoimmune hepatitis or toxic hepatitis; or (5) refusal or inability to participate in the investigation because of critical health. To identify and exclude participants with HBV infection from the present study, serological markers (including hepatitis B surface antigen [HBsAg], total hepatitis B core antibody [anti-HBc], and hepatitis B surface antibody [anti-HBs]) for HBV infection were tested using commercial enzyme-linked immunosorbent assay kits (Wantai BioPharm, Beijing, China). Participants with positive serology for HBsAg were excluded from further investigation. To be more accurate, subjects were included if any of the following serological markers statuses were observed: (1) HBsAg-negative, anti-HBc-negative, and anti-HBs-negative; (2) HBsAg-negative, anti-HBc-negative, and anti-HBs-positive; or (3) HBsAg-negative, anti-HBc-positive, and anti-HBs-positive. Therefore, five study subjects with HBsAg-negative, anti-HBc-positive, and anti-HBs-positive were eligible for inclusion in the study due to the immunity triggered by natural infection. To determine HCV infection, viral RNA was extracted from 650-µL serum samples using a CobasAmpliPrep automated extractor (Roche Molecular Systems, Pleasanton, CA, USA) according to the manufacturer’s instructions. The CobasTaqMan 96 analyzer (Roche Molecular Systems) was used for automated real-time polymerase chain reaction (PCR) amplification and detection of PCR products. Data were analyzed using AMPLILINK software, version 3.3 (Roche Molecular Systems).

Each healthy control was pair-matched by gender and age within 3 years to an eligible case with HCC. Control subjects were Han Chinese residents of Xiamen City without prior histories of HCC, liver cirrhosis, or HBV or HCV infection. In order to minimize risk of bias, we randomly selected control subjects from the National Nutrition Survey and confirmed that all study subjects were unrelated by blood. Overall, 220 HCC cases and an equal number of healthy controls were enrolled in our study.

A questionnaire was used to collect information on demographic characteristics, family cancer history, food consumption, lifestyle, and environmental factors from all study subjects. Interviews were performed by extensively trained staff to improve data quality and to minimize inter-interviewer variation. All cases and healthy controls provided a 5-mL blood sample on the day of the interview. Blood samples were centrifuged at 4000 rpm for 10 min to separate plasma and blood cells and were then frozen at −78°C prior to DNA extraction. All study subjects provided written informed consent, in agreement with the Helsinki declaration and the policy of the Ethics Committee of Xiamen Center for Disease Control and Prevention, which approved this study.

### DNA extraction and genotyping

Human genomic DNA was extracted from blood cells using the MagNA Pure LC DNA Isolation Kit I (Roche Applied Science, Mannheim, Germany) and stored at −22°C in screw-capped tubes. All samples were genotyped using the Sequenom platform in accordance with the manufacture’s iPLEX Application Guide (Sequenom, San Diego, CA, USA). Initial PCR amplification was performed using the GeneAmp PCR system 9700 thermal cycler (Applied Biosystems) in a total volume of 5 µL with 10 ng of genomic DNA, 3.5 mM of MgCl_2_, 0.5 U of HotStarTaq polymerase (Qiagen, Valencia, CA, USA), 500 µM of dNTPs (Invitrogen, Carlsbad, CA, USA), and 60 nM of each primer set. Amplification was performed as follows: denaturation at 94°C for 15 s followed by 45 cycles of 94°C for 20 s, 56°C for 30 s, 72°C for 1 min, and 72°C for 3 min. Subsequently, PCR products were treated with a mixture of 1.53 µL H_2_O, 0.17 µL 10× Shrimp Alkaline Phosphatase (SAP) Buffer, and 0.3 µL SAP (1.7 U/µL) at 37°C for 40 min and 85°C for 5 min. The SAP-treated products were then subject to iPLEX extension, during which time the primers hybridized to their target regions, which were extended by a single mass-modified nucleotide. The final extension reaction contained 0.222 µL iPLEX buffer plus (10×), 0.2 µL iPLEX termination mix, 0.041 µL iPLEX enzyme, 0.619 µL H_2_O, and 0.940 µL of iPLEX extend primer mix at optimized concentrations (Sequenom). The working conditions for single base extension were as follows: 94°C for 30 s, followed by a total of 200 nested PCR cycles comprising 40 main cycles of 94°C for 5 s, 5 sub cycles of 52°C for 5 s and 80°C for 5 s, and a final extension for 3 min at 72°C. To minimize background noise, iPLEX reaction products were treated with cationic exchange resin for 30 min to remove salts. At the end of the experiment, samples were spotted on SpectroCHIP arrays (Sequenom) using a MassArray Samsung Nanodispenser and scanned using a matrix-assisted laser desorption-ionization time-of-flight mass spectrometry system. Genotyping results were analyzed using MassArrayTyper 4.0 (Sequenom) software. A negative water control and reference DNA were employed as quality control measures during the genotyping assay. In addition, approximately 5% of the samples were randomly selected and repeated for genotyping as duplicated controls. As a result, the genotyping call rate was 100%.

### Data collection and statistical analysis

Eligible subjects underwent face-to-face interviews to obtain demographic data, which were recorded with double-entry verification using EpiData version 3.1 (The EpiData Association, Odense, Denmark). Statistical analysis was performed using IBM Statistics SPSS version 19 (Armonk, NY, USA). Initially, we compared demographic data between HCC cases and controls using a two-sided Chi-square test to identify differences between the groups. Hardy-Weinberg equilibrium (HWE) for *MBL2* rs7096206 was calculated using an online calculator with an alpha of 0.05. To assess the effect of *MBL2* rs7096206 on HCC risk, odds ratios (ORs) and 95% confidence intervals (CIs) were estimated using conditional logistic regression analysis.

## RESULTS

### Subject characteristics

Demographic characteristics of all study subjects are shown in Table 1. Age, gender, and ethnicity were fully matched between cases and controls, and there were no significant differences between cases and controls with respect to education (*P* = 0.06) or marital status (*P* = 0.83). There were also no significant differences between groups in alcohol consumption (*P* = 0.59) or smoking status (*P* = 0.22).

**Table 1.** Demographic characteristic of hepatocellular carcinoma cases and controls

Variable	Cases (*n* = 220)		Controls (*n* = 220)		*P*
	
	*n*	%	*n*	%	
Age distribution (years)					
≤30	3	1.4	3	1.4	
31–50	45	20.5	45	20.5	
51–70	126	57.3	126	57.3	
≥71	46	20.9	46	20.9	1
Gender					
Male	188	85.5	188	85.5	
Female	32	14.5	32	14.5	1
Education level					
Elementary	135	61.4	128	58.2	
Middle/High school	50	22.7	45	20.5	
College or higher	35	15.9	47	21.3	0.06
Marital status					
Married	161	73.2	166	75.5	
Divorced	12	5.5	10	4.5	
Widowed	41	18.6	36	16.3	
Single	6	2.7	8	3.7	0.83
Alcohol consumption					
None	49	22.3	40	18.2	
Low (1–2 times/week)	50	22.7	45	20.5	
Medium (3–4 times/week)	72	32.7	80	36.4	
High (more than 4 times/week)	49	22.3	55	25.0	0.59
Smoking status					
Nonsmokers	65	29.6	77	35.0	
Smokers	155	70.4	143	65.0	0.22

### *MBL2* rs7096206 and HCC risk

Table 2 shows the genotype distributions of *MBL2* rs7096206 between HCC cases and controls. The genotype distribution was in HWE in the control group (*P* > 0.05), suggesting that there was no sampling bias. Both the homozygote (GG) and heterozygote (CG) variants demonstrated a significantly elevated risk of developing HCC. The odds of HCC among carriers of CG and GG genotypes were estimated as 7.33 (95% CI, 2.53–21.29) and 12.48 (95% CI, 2.08–74.90), respectively, compared to wild type carriers. In the dominant model, GG+CG carriers had an approximately 8-fold increase in risk (95% CI, 2.83–22.62) compared with those with the CC genotype. GG carriers possessed a 4.5-fold increased risk (95% CI, 0.97–20.83) in the recessive model, but this was not significant. The G allele was significantly positively associated with an elevated HCC odds ratio of 6.83 (95% CI, 2.90–16.10) compared to those without the G allele.

**Table 2.** Association between MBL2 polymorphism and risk of HCC

Genotypes	Cases	Controls	OR (95% CI)^a^	*P*
*rs7096206 (MBL2)*				
CC	125	153	1 (Ref.)	—
CG	86	65	7.33 (2.53–21.29)	*0.000*
GG	9	2	12.48 (2.08–74.90)	*0.006*
Dominant Model				
CC	125	153	1 (Ref.)	—
GG+CG	95	67	8.00 (2.83–22.62)	0.000
Recessive Model				
CC+CG	211	218	1 (Ref.)	—
GG	9	2	4.50 (0.97–20.83)	0.054
Allele				
C	336	371	1 (Ref.)	—
G	104	69	6.83 (2.90–16.10)	0.000

CI, confidence interval; OR, odds ratio; HCC, hepatocellular carcinoma; MBL2, mannose-binding lectin 2.

^a^Calculated using a conditional regression model.

## DISCUSSION

An investigation by the Center for Disease Control and Prevention and the World Health Organization revealed that most global HCC incidence is attributable to infection by HBV (53%) or HCV (25%).^8^ However, a relatively large proportion of Chinese HCC cases occur in patients free from HBV infection. Exposure to aflatoxin B1^9^ and pesticides^10^ and high concentrations of iron in drinking water^11^ have also been shown to be associated with HCC risk, although there is increasing evidence for genetic susceptibility.

The complement system plays many roles in host immunity and defense against infection. Its activation leads to opsonization of pathogens and immune complexes, the recruitment of leukocyte inflammation, and cellular lysis.^12^ The lectin pathway is activated through the recognition of pathogens by MBL and ficolins,^13^ after which circulating MBL forms complexes with the MBL-associated serine proteases mannan-binding lectin serine protease (MASP)-1, MASP-2, and MASP-3.^14^ The association with MASP-2 yields a complex that triggers complement activation through cleavage of components C4 and C2.^15^

Polymorphisms in *MBL* genes were shown to be involved in the host immune response against HBV infection,^16^ suggesting that subjects with low serum MBL levels are more likely to become infected with HBV. However, another study found no significant correlation between *MBL* polymorphisms and chronic HBV infection.^17^ Similarly, limited data is available on the association between *MBL2* polymorphisms and HCC risk. A case-control study conducted in an Italian population found no significant association between *MBL2* SNPs and HCC risk. We re-analyzed these data and found the OR of HBV- and HCV-negative variant homozygotes to be 0.85 (95% CI, 0.10–7.08) and 0.87 (95% CI, 0.35–2.14),^7^ respectively. There was no significant difference in MBL2 polymorphism genotype distribution between HBV-positive HCC cases and healthy controls. However, this study was limited by its small sample size; moreover, the HBV/HCV infection status of the controls was unavailable, so the risk may not have been fully assessed.

In the present study, we found that both rs7096206 CG and GG genotypes were significantly correlated with elevated HCC risk, with respective ORs of 7.33 (95% CI, 2.53–21.29) and 12.48 (95% CI, 2.08–74.90). This significance was also observed under the dominant genetic model but not the recessive one. Moreover, the G allele conferred a 6.83-fold increased risk of HCC compared with the C allele. Compared with previous work, our study had a larger sample size and cancers caused by HBV or HCV infection were ruled out in the analysis, so calculation of the ORs was not confounded by viral infection. These findings suggested that *MBL2* SNP rs7096206 is associated with increased HCC risk in a hepatitis-free population and that it has the potential to serve as a biomarker for detecting populations at increased HCC risk. We also propose that the role of MBL2 in HCC development should be further investigated.
